# Functional Deficits in Non-elite Soccer (Football) Players: A Strength, Balance, and Movement Quality Assessment After Anterior Cruciate Ligament Reconstruction

**DOI:** 10.7759/cureus.75846

**Published:** 2024-12-17

**Authors:** Maciej Biały, Bartosz Wilczyński, Florian Forelli, Timothy E Hewett, Rafał Gnat

**Affiliations:** 1 Department of Physiotherapy, Institute of Physiotherapy and Health Sciences, The Jerzy Kukuczka Academy of Physical Education, Katowice, POL; 2 Department of Physiotherapy, Functional Diagnostics Laboratory, Sport-Klinika, Scanmed Sport, Żory, POL; 3 Department of Immunobiology and Environmental Microbiology, Medical University of Gdańsk, Gdańsk, POL; 4 Department of Sports Rehabilitation, Orthosport Rehab Center, Domont, FRA; 5 Department of Orthopaedic Surgery, Clinic of Domont Ramsay Healthcare, Domont, FRA; 6 Research Unit, Société Française des Masseurs Kinésithérapeutes du Sport (SFMKS) Lab, Pierrefitte-sur-Seine, FRA; 7 Department of Orthopaedic Surgery, Marshall University Joan C. Edwards School of Medicine, Huntington, USA

**Keywords:** acl reconstruction, balance, functional movement screen, isokinetic strength, neuromuscular recovery, non-elite soccer players, return to sport

## Abstract

Introduction: Anterior cruciate ligament (ACL) reconstruction (ACLR) is the gold standard for treating ACL injuries, particularly in soccer players who are at a high risk of knee injury. While professional athletes often return to sport (RTS) within 7-10 months after ACLR, non-elite players experience significant delays. There is a need to investigate neuromuscular deficits and functional asymmetries in the non-elite group, which may persist even after clearance for RTS. This study aims to evaluate the functional movement patterns, single-leg balance, and isokinetic knee strength in non-elite soccer players preparing to RTS. Additionally, correlations between the aforementioned parameters were explored.

Methods: A cohort of 69 male, non-elite soccer players (mean age: 24.32±8.56 years) who underwent ACLR with a hamstring graft was recruited. Functional assessments were conducted at the mean time of nine months post-surgery, and all participants were clinically cleared by the surgeon. The evaluation included the Functional Movement Screen™ (FMS), single-leg balance tests (SLBT) on stable and unstable platforms, and isokinetic strength tests (IST) for knee flexion and extension at 60 deg·s^-1^ and 180 deg·s^-1^. Limb symmetry indices (LSIs) were calculated. Statistical analyses included t-tests, Mann-Whitney U tests, and Spearman correlations.

Results: The mean FMS score was 15.45±2.23, indicating moderate functional movement quality. SLBT results revealed no significant (P>0.05) inter-extremity differences in stability indices, regardless of platform setting (stable or unstable). However, significant deficits in quadriceps and hamstring strength were observed in the ACLR extremity. At 60 deg·s^-1^, the mean peak torque/body weight (PT/BW) ratio for knee extension was 2.01±0.65 Nm.kg^-1^ for the ACLR side versus 2.60±0.57 Nm.kg^-1^ for the contralateral side (P<0.0001). Similar asymmetries were observed at 180 deg·s^-1^ (1.51±0.44 vs. 1.88±0.35 Nm.kg^-1^; P<0.0001). LSIs for quadriceps were markedly reduced, averaging 76.97±17.72% at 60 deg·s^-1^ and 79.89±17.11% at 180 deg·s^-1^. At 60 deg·s^-1^, the mean PT/BW ratio for knee flexion was 1.24±0.34 Nm.kg^-1^ for the ACLR side versus 1.39±0.32 Nm.kg^-1^ for the contralateral side (P=0.009) at 180 deg·s^-1^ (1.03±0.27 vs. 1.16±0.25 Nm.kg^-1^; P=0.003). LSIs for the hamstring were averaging 89.34±13.91% at 60 deg·s^-1^ and 88.44±14.58% at 180 deg·s^-1^. Weak negative correlations were found between FMS scores and stability indices (e.g., r=-0.26 for overall stability index on unstable platform; P=0.031), while moderate positive correlations were observed between PT/BW ratios and FMS scores (r=0.36-0.60; P<0.001).

Conclusions: Significant strength asymmetries in quadriceps and hamstring muscles persist in non-elite soccer players after ACLR. While balance deficits were minimal, weak correlations between FMS scores and stability indices suggest a link between functional movement quality and dynamic control. These findings highlight the need for targeted strength training in rehabilitation and the importance of comprehensive assessments, including functional performance tests, strength evaluations, and balance analysis, to ensure safe RTS. Achieving clinical clearance does not equate to full recovery, emphasizing the necessity for a multidimensional approach to RTS decisions.

## Introduction

A growing number of people worldwide participate in soccer (football) at both amateur and professional levels. According to the 2023 report of the International Federation of Association Football (FIFA), there are currently 128,694 professional soccer players globally [1]. Additionally, the estimated non-elite soccer-playing population stands at approximately 260 million. One of the primary concerns for soccer players is the risk of serious knee injury [2], with anterior cruciate ligament (ACL) injuries being among the most common and increasing among these athletes at an average rate of 6% per year [3]. Interestingly, ACL injuries occur more frequently in amateurs, with an incidence of 0.074 per 1,000 hours of play, as compared to 0.058 per 1,000 hours in the professional group [4]. This type of injury not only impairs sports performance and reduces quality of life but can also lead to withdrawal from full sports participation [5]. When considering treatment options for ACL rupture, ACL reconstruction (ACLR) remains the gold standard [6]. Ardern et al.'s systematic review found that 63% of athletes returned to their pre-injury level of sport after ACL rupture, while only 44% of competitive athletes achieved the same level of participation [7]. Regarding failure and revision rates, the estimated overall ACL re-injury rate among athletes equals 15% [8].

The functional recovery of non-elite soccer players may not receive the same level of attention as that of elite players. While 80-97% of professionals return to play within 7-10 months after ACLR [3], only around 50% of amateurs return to the field two years after ACLR [9]. Moreover, competitive players exhibit higher strength levels and better self-reported scores as compared to amateurs [10]. However, the latest study conducted by Annibaldi et al. found that among amateur players who underwent ACLR, 73.8% returned to playing soccer, with 93.5% resuming play at their pre-injury level. Despite these promising numbers, the overall failure rate was 16%, predominantly affecting athletes aged 21 years or younger [11]. According to Chatzilamprinos et al., two years after ACLR, soccer players continue to show strength and functional performance deficits, with significant weakness in peak torque (PT) of knee extensors in the operated extremity, as compared to the healthy one [12]. These outcomes vary depending on the assessment period, recovery timelines, and performance outcomes between elite and non-elite athletes.

Previous research indicates that multiple risk factors contribute to ACL re-injury, including graft type, surgical procedure, demographic factors such as sex and age, activity level, and deficits or asymmetries in neuromuscular and biomechanical function, which can persist up to two years after ACLR [13]. To reduce the risk of re-injury following ACLR, addressing neuromuscular deficits and functional asymmetries is essential, and the assessment of the lower extremity muscle strength, balance, and movement patterns is crucial, regardless of the patient's level of sports activity and time frames after surgery. This study aims to bridge the gap in the literature by evaluating the functional movement patterns expressed by the outcomes of the Functional Movement Screen (FMS), single-leg balance test (SLBT), and isokinetic strength test (IST) in non-elite soccer players examined at the critical time-point, i.e., cleared by the surgeon, and preparing to return to sport (RTS). By focusing on this group, we aim to highlight the unique challenges they face and emphasize the importance of functional assessment as a foundation for full recovery. Additionally, we verify the correlation between tested variables, thereby offering insights that could enhance rehabilitation protocols and injury prevention strategies.

## Materials and methods

Data used in the study were collected from a group of patients at Sport-Klinika, Żory, Poland, between 2013 and 2020. This study is based on a retrospective cohort design with a purposive sampling strategy after obtaining approval from the Bioethics Committee at the Silesian Chamber of Physicians (approval number: ŚIL.KB.711.2022.MP). All participants gave their written informed consent. The functional assessment included the FMS, SLBT, and IST. These tests were performed after participants were clinically cleared by the surgeon (general knee joint evaluation with laxity testing) and referred for functional evaluation before RTS. The mean time from ACLR to measurement was 9.04±1.09 months.

Sixty-nine patients (male, non-elite soccer players, individuals participating in amateur or recreational soccer leagues without professional contracts; for demographic characteristics, see Table 1) who underwent primary ACLR were recruited. The surgical procedures were completed by two surgeons with at least 15 years of experience in ACL surgery. All participants met the following inclusion criteria: diagnosis of ACL rupture without any other injuries confirmed by magnetic resonance imaging, followed by ACLR with hamstring graft. Participants were excluded if they had a history of previous ACLR or any different procedures including meniscus repair, partial meniscectomy, or cartilage repair as well in ACLR as in the contralateral extremity. Additionally, individuals who participated in soccer on more than amateur level or those who did not attend regular soccer training at least twice a week, with performance in local leagues and tournaments, were not included. None of the participants underwent preoperative rehabilitation. The rehabilitation protocol following ACLR was divided into four progressive phases. Phase 1 (weeks 1-2) focused on alleviating pain, reducing stiffness, managing edema, and initiating isometric quadriceps activation. Phase 2 (weeks 3-6) emphasized improving range of motion, normalizing gait, and introducing closed kinematic chain exercises such as squats. Phase 3 (weeks 7-12) aimed to restore symmetrical knee mobility, develop strength, and incorporate running, cycling, and jumping drills. Finally, phase 4 (weeks 13-26) progressed to advanced strength training.

**Table 1:** Demographic characteristics of the study group. ACLR: anterior cruciate ligament reconstruction

	Mean±SD (min-max)/N (%)
Number	69
Gender	Male: 69 (100)
Age (y)	24.32±8.56 (14-49)
Body mass (kg)	78.03±12.25 (56-119)
Body height (cm)	178.29±7.54 (163-195)
Injury to ACLR (mo)	12.27±15.55 (1-65)
ACLR to measurement (mo)	9.04±1.09 (6-13)

The measurements were conducted by two certified physiotherapists, each with over 10 years of orthopedic physiotherapy experience and five years of specialized training in functional testing. To ensure objectivity, a third independent rater processed the data without any knowledge of the study objective. Measurement reliability was confirmed prior to the assessment in 12 healthy subjects, with a weighted kappa coefficient of 0.75 for FMS. Additionally, intra-rater reliability was assessed, yielding an intra-class correlation coefficient (ICC) of 0.88 for SLBT and 0.94 for the PT/body weight (PT/BW) ratio in the IST. ICC values are interpreted according to established criteria as follows: <0.5 (poor reliability), 0.5-0.75 (moderate reliability), 0.75-0.9 (good reliability), and >0.90 (excellent reliability) [14].

Before the examination, each participant completed a 10-minute warm-up session on a bicycle ergometer. During this period, the rater provided a brief overview of the functional assessment procedures. Patients were encouraged to report any discomfort or pain experienced during the assessment and could choose to stop the tests at any time. Individuals experiencing pain or discomfort in the musculoskeletal system, as indicated by a >0 score on the Visual Analogue Scale on the day of measurement, were excluded to ensure the accuracy and relevance of the study findings.

The functional assessment was initiated with standardized FMS tests, consisting of seven dynamic exercises to evaluate fundamental movement patterns. These tests assessed various factors, including muscle strength, flexibility, range of motion, and neuromuscular control, to detect significant motor dysfunctions or asymmetries [15]. Each participant performed three trials of the seven FMS tests: deep squat, in-line lunge, hurdle step, shoulder mobility, active straight leg raise, trunk stability push-up, and quadruped rotary stability. Participants were scored on a scale of 0-3 points per test based on specific criteria: 0 (for pain during movement), 1 (for inability to complete the task), 2 (for task completion with compensatory movements), and 3 (for efficient task completion). The highest possible total score was 21 points. The FMS is considered as a reliable tool for musculoskeletal screening [16].

The SLBT was performed using the Biodex Balance System (Biodex Medical Systems, Shirley, NY, USA), which utilizes sensors beneath the platform to measure deviations in the subject's center of pressure. The assessment included static (platform locked) and dynamic (platform tilting up to 20 degrees) conditions, with the dynamic measurements conducted at level 4, for safety reasons. The SLBT provided three outcome measures, overall stability index (OSI), anterior-posterior (AP) index, and medial-lateral (ML) index, representing the standard deviations of the platform's inclination angle. For static tests, the device calculated the projection of the center of pressure, determining the presumed angle of platform tilt. Lower scores, indicating less sway, were more desirable [17]. The OSI, AP, and ML indices were derived using the formula described by Arnold and Schmitz [17]. During the test, participants assumed a one-legged stance on the platform with the contralateral extremity flexed at 90 degrees at the knee joint. Each subject completed three 15-second repetitions without visual feedback, and the average values of each index were used for the analysis.

Following a five-minute rest period, measurements of quadriceps and hamstring muscle strength using the Biodex System 4 (Biodex Medical Systems, Shirley, NY, USA) took place. PT during isokinetic knee flexion and extension were evaluated at the angular velocities of 60 deg·s^-1^ and 180 deg·s^-1^. Participants were seated in the dynamometer and stabilized with belts around the trunk, pelvis, and thighs, with the resistance pad placed at the level of the medial malleolus of the tested extremity. A one-minute rest was provided between trials. For each muscle group and velocity, PT was normalized to BW (PT/BW ratio (Nm.kg^-1^)). The IST have shown high test-retest reliability (ICC ranging from 0.81 to 0.97) in patients after ACLR [18]. Limb symmetry indices (LSIs) for knee flexion and extension were also calculated for both velocities using the formula LSI=(PT/BW of the injured extremity/PT/BW of the uninjured extremity)×100%.

The statistical analysis was performed using the Statistica 13.0 software (StatSoft, Tulsa, OK, USA). Deviations from the normal distribution were tested using the Shapiro-Wilk test and homogeneity of variances using the Levene test. Due to the frequent significant outcomes of the Shapiro-Wilk test, the statistical analyses of differences were partially based on the parametric independent Student's t-test and partially on the nonparametric Mann-Whitney U test. The lower extremity (ACLR vs. contralateral) was regarded as the independent factor in such analyses. The critical P level was set at 0.05. Effect sizes were presented as Cohen's d coefficients calculated using Z statistics for the Mann-Whitney U test and mean values/standard deviations for Student's t-test with values of 0.2, 0.5, and 0.8 interpreted as small, medium, and large effects, respectively.

## Results

**Figure 1:**
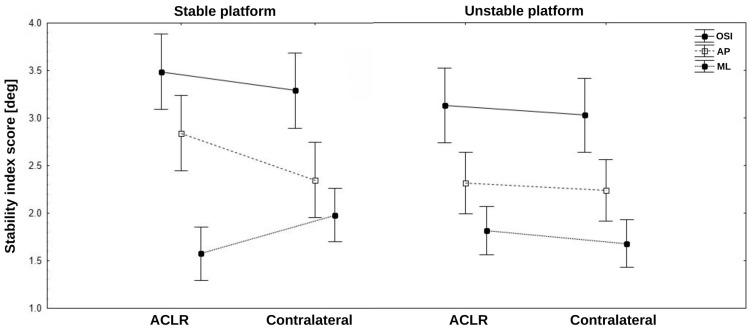
Mean values±95% confidence intervals (whiskers) of the overall, AP, and ML stability indices recorded on the stable and unstable platforms for the operated (ACLR) and contralateral extremities. Inter-extremity differences are not significant. OSI: overall stability index; AP: anterior-posterior; ML: medial-lateral; ACLR: anterior cruciate ligament reconstruction

In the study group, the mean total score of the FMS test was 15.45±2.23, ranging from eight to 19 points. Across all recorded stability indices (OSI, AP, ML), no significant differences were observed between extremities, regardless of platform setting (stable or unstable). On the stable platform, the mean stability indices recorded were as follows: OSI was 3.49±1.70 for the ACLR and 3.29±1.63 for the contralateral (P=0.5; Cohen's d=1.1), AP was 2.84±1.87 for the ACLR and 2.35±1.31 for the contralateral (P=0.2; Cohen's d=0.22), and ML was 1.57±0.96 for the ACLR and 1.98±1.37 for the contralateral (P=0.12; Cohen's d=0.27). For the unstable platform, the mean indices were slightly lower: OSI was 3.13±1.73 for the ACLR and 3.03±1.55 for the contralateral (P=0.57; Cohen's d=0.1), AP was 2.32±1.41 for the ACLR and 2.24±1.31 for the contralateral (P=0.84; Cohen's d=0.04), and ML was 1.82±1.13 for the ACLR and 1.68±0.98 for the contralateral (P=0.37; Cohen's d=0.16). Overall, there was a slight trend for participants to demonstrate lower stability scores for the contralateral extremity, with the exception of the ML index on the stable platform. These findings are illustrated in Figure 1 and detailed in Table 2.

**Table 2:** Mean values±standard deviations (minima-maxima) of the overall, AP, and ML stability indices (in degrees) recorded on the stable and unstable platforms for the operated (ACLR) and contralateral lower extremities. Also, presented are P levels (Cohen's d effect sizes) for the inter-extremity differences (Mann-Whitney U test). OSI: overall stability index; AP: anterior-posterior; ML: medial-lateral; ACLR: anterior cruciate ligament reconstruction

Platform	Index	ACLR	Contralateral	P (Cohen's d)
Stable	OSI	3.49±1.70 (0.90-8.40)	3.29±1.63 (0.70-8.80)	0.50 (0.11)
	AP	2.84±1.87 (0.60-8.40)	2.35±1.41 (0.50-6.70)	0.20 (0.22)
	ML	1.57±0.96 (0.40-3.80)	1.98±1.37 (0.40-6.90)	0.12 (0.27)
Unstable	OSI	3.13±1.73 (0.90-12.30)	3.03±1.55 (0.70-9.10)	0.57 (0.10)
	AP	2.32±1.41 (0.70-7.10)	2.24±1.31 (0.60-7.20)	0.84 (0.04)
	ML	1.82±1.13 (0.30-8.10)	1.68±0.98 (0.40-5.20)	0.37 (0.16)

**Figure 2:**
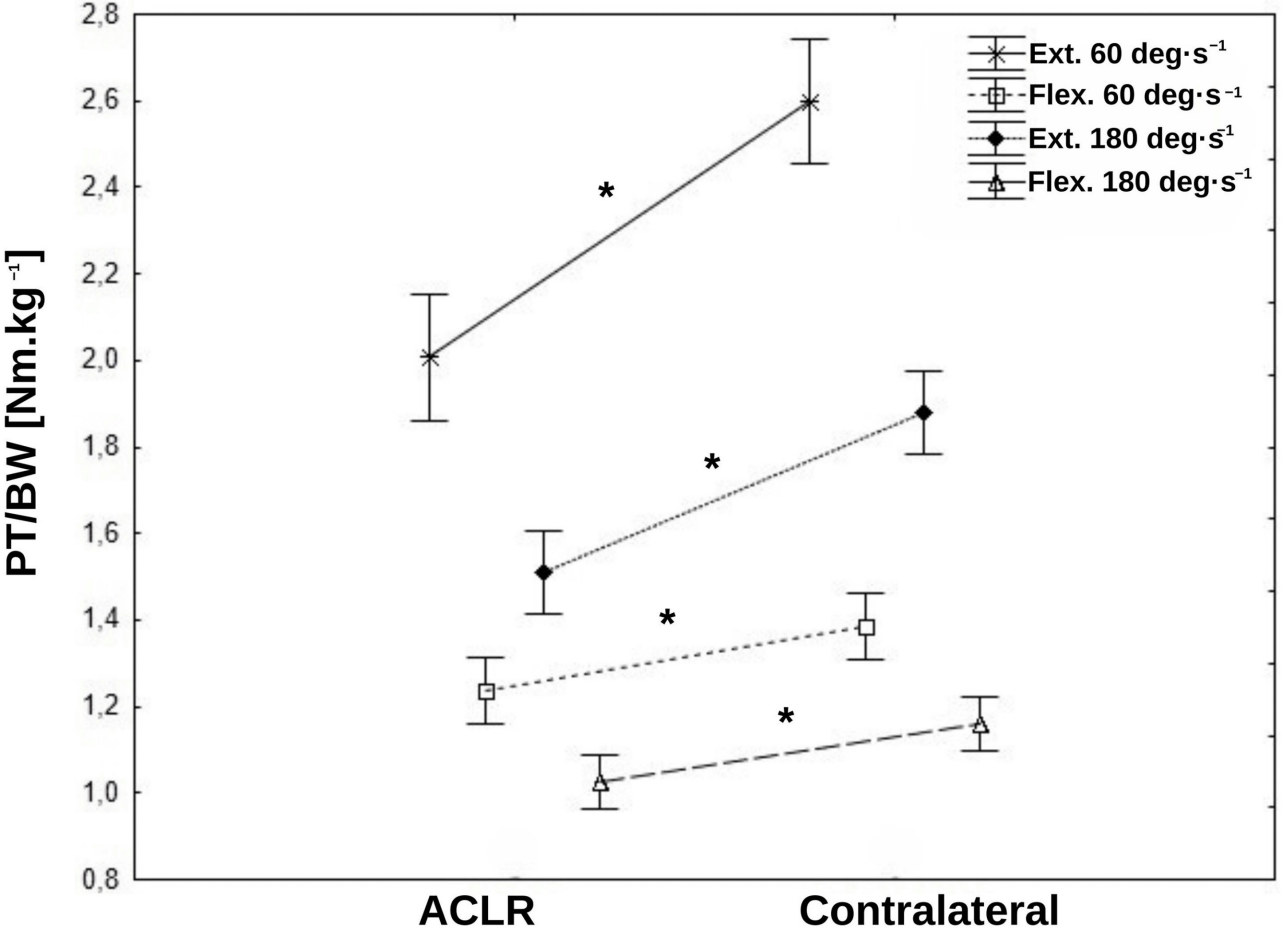
Mean values±95% confidence intervals (whiskers) of the PT/BW ratio (in Nm/kg) recorded for the operated (ACLR) and contralateral lower extremities during movements of extension and flexion of the knee joint with angular velocities of 60 and 180 deg•s-1. All inter-extremity differences are significant. ACLR: anterior cruciate ligament reconstruction; PT/BW: peak torque/body weight

Significant inter-extremity differences were observed across all IST for the PT/BW ratios (Figure 2, Table 3). These differences were especially pronounced during knee extension (quadriceps strength) at both tested angular velocities. At 60 deg·s^-1^, the mean PT/BW ratio for the ACLR was 2.01±0.65 Nm.kg^-1^ and 2.60±0.57 Nm.kg^-1^ for the contralateral (P<0.0001; Cohen's d=0.97). At 180 deg·s^-1^, these values were 1.51±0.44 Nm.kg^-1^ for the ACLR and 1.88±0.35 Nm.kg^-1^ for the contralateral (P<0.0001; Cohen's d=0.93). While differences were less marked in knee flexion (hamstring strength), they remained statistically significant. At an angular velocity of 60 deg·s^-1^, the PT/BW ratios were 1.24±0.34 Nm.kg^-1^ for the ACLR and 1.39±0.32 Nm.kg^-1^ for the contralateral (P=0.009; Cohen's d=0.45). At 180 deg·s^-1^, the mean values were 1.03±0.27 Nm.kg^-1^ for the ACLR and 1.16±0.25 Nm.kg^-1^ for the contralateral (P=0.003; Cohen's d=0.52). A clear inter-extremity asymmetry was observed across all recorded PT/BW ratios. For quadriceps strength, the mean LSI at an angular velocity of 60 deg·s^-1^ was 76.97±17.72%, with a range of 32.05-122.02. At a velocity of 180 deg·s^-1^, the LSI increased slightly to 79.89±17.11%, ranging from 35.55 to 115.82. For hamstring strength, the LSI values were generally higher: at 60 deg·s^-1^, the mean was 89.34±13.91%, with a range of 36.65-117.03, and at 180 deg·s^-1^, it was 88.44±14.58%, with values ranging from 62.50 to 120.32.

**Table 3:** Mean values±standard deviations (minima-maxima) of the peak torque to body weight ratio (in Nm/kg) recorded for the operated (ACLR) and contralateral lower extremities during movements of extension and flexion of the knee joint. Also, presented are P levels (Cohen's d effect sizes) for the inter-extremity differences (independent Student's t-tests). *statistically significant ACLR: anterior cruciate ligament reconstruction

Velocity	Movement	ACLR	Contralateral	P (Cohen's d)
60 deg·s^-1^	Extension	2.01±0.65 (0.56-3.42)	2.60±0.57 (1.27-4.07)	<0.0001 (0.97)*
	Flexion	1.24±0.34 (0.41-2.09)	1.39±0.32 (0.58-2.11)	0.009 (0.45)*
180 deg·s^-1^	Extension	1.51±0.44 (0.57-2.38)	1.88±0.35 (0.95-2.61)	<0.0001 (0.93)*
	Flexion	1.03±0.27 (0.49-1.65)	1.16±0.25 (0.58-1.66)	0.003 (0.52)*

In the analysis of correlations, weak negative correlations were found between the FMS total score and all stability indices for the ACLR extremity. Three of them proved significant (FMS total score and OSI on stable platform (r=-0.25; P=0.040), OSI on unstable platform (r=-0.26; P=0.031), and ML stability index on unstable platform (r=-0.26; P=0.028)). In the contralateral extremity, these correlations were both positive and negative. Only one significant outcome was recorded (ML stability index on unstable platform (r=-0.25; P=0.039)). For all IST, positive significant correlations of moderate strength were revealed (r-Spearman 0.36-0.60; all P≤0.003). LSIs showed moderate, positive, and significant correlations with FMS composite score only for the movement of knee extension at the angular velocity of 60 deg·s^-1^ (r=0.45; P<0.0001) and of 180 deg·s^-1^ (r=0.44; P<0.0001). Detailed information is presented in Table 4.

**Table 4:** R-Spearman correlation coefficients (P level of correlation) for correlations between Functional Movement Screen total score and stability indices (overall, AP, ML), PT/BW ratios, and LSI. *statistically significant OSI: overall stability index; AP: anterior-posterior; ML: medial-lateral; ACLR: anterior cruciate ligament reconstruction; PT/BW: peak torque/body weight; LSI: limb symmetry index

Functional test	Variable	ACLR	Contralateral	Both extremities
Stable platform	OSI	-0.25 (0.040)*	0.09 (0.444)	-
	AP	-0.22 (0.074)	0.02 (0.902)	-
	ML	-0.06 (0.606)	0.04 (0.734)	-
Unstable platform	OSI	-0.26 (0.031)*	-0.08 (0.530)	-
	AP	-0.21 (0.081)	0.05 (0.706)	-
	ML	-0.26 (0.028)*	-0.25 (0.039)*	-
PT/BW	Extension 60 deg·s^-1^	0.60 (<0.0001)*	0.43 (<0.0001)*	-
	Flexion 60 deg·s^-1^	0.48 (<0.0001)*	0.47 (<0.0001)*	-
	Extension 180 deg·s^-1^	0.60 (<0.0001)*	0.43 (<0.0001)*	-
	Flexion 180 deg·s^-1^	0.36 (0.003)*	0.50 (<0.0001)*	-
LSI	Extension 60 deg·s^-1^	-	-	0.45 (<0.0001)*
	Flexion 60 deg·s^-1^	-	-	0.10 (0.397)
	Extension 180 deg·s^-1^	-	-	0.44 (<0.0001)*
	Flexion 180 deg·s^-1^	-	-	-0.06 (0.609)

## Discussion

The study provides insights into the functional performance, lower extremity balance control, and muscle strength of non-elite soccer players after ACLR who had all been clinically cleared for RTS. The data have been presented with regard to measurement reliability and effect sizes for all registered differences. The recorded FMS total scores indicate a moderate level of functional movement quality in participants. No statistically significant differences were found between lower extremity stability indices. In contrast, the IST revealed significant inter-extremity differences in quadriceps and hamstring muscle strength, as expressed by PT/BW ratios and estimated LSI values.

It has to be underlined that comparisons of FMS scores in ACLR athletes show varied outcomes across studies [19-24]. The FMS total score in our study group was 15.45±2.23 (range: 8-19 points). According to our unpublished data, the reference FMS values for a control group of uninjured, non-elite athletes are 15.80±1.80 (range: 12-20 points), with no statistically significant difference observed between the mean total scores of our study group and controls (independent t-test, P=0.156). Within our study group, 51% of participants scored below the reference standard (15 points), while 49% scored above this threshold. Similarly, Mayer et al. [19] found no significant FMS total score differences between athletes cleared for RTS (12.7±2.9 points) and those not cleared (12.8±2.7 points). Moreover, in the study of 35 amateur soccer players six months after ACLR, Kublin et al. recorded an FMS total score of 15.34±2.60, aligning closely with our control group from unpublished data [20]. In contrast, Oleksy et al. reported that soccer players after ACLR had significantly lower FMS total score (12±4 points) compared to a control group of uninjured players (15±2 points) [21]. In a previous study, where we explored the FMS ability to detect asymmetries in non-elite athletes at the early stage after ACLR (3.4±0.5 months postoperatively), our finding was that early assessments may overestimate functional capacity, with an average FMS total score of 14.7±2.4 [22]. In interpreting whether FMS scores are adequate for RTS, the study by Shojaedin et al. provides some insights, as it indicates that athletes with pre-season FMS scores below 17 points are 4.7 times more likely to sustain lower extremity injuries, reinforcing the predictive value of the FMS in assessing injury or re-injury risk [23]. The evidence suggests that meeting RTS benchmarks does not imply complete neuromuscular recovery; athletes may achieve adequate FMS scores through compensatory movement patterns that mask deficits, which could increase the risk of re-injury [24]. These findings point out the need for cautious interpretation of FMS scores in athletes after ACLR, emphasizing the importance of considering various factors, such as the timing of assessment after ACLR, potential compensatory patterns, and the athlete's level of sports activity, in comprehensive RTS decision-making.

It has been proven that soccer players undergoing ACLR exhibit impaired single-leg stance and postural control, which can persist long-term following surgery [25]. In our study, SLBT results revealed no statistically significant differences in any of the measured stability indices between the ACLR and contralateral extremity, regardless of the platform setting. On the stable platform, the ACLR extremity showed slightly higher values, except for the ML index, which displayed an opposite trend. On the unstable platform, stability values were generally lower, reflecting increased postural demands and suggesting the use of compensatory mechanisms to maintain dynamic control in the ACLR extremity. However, the differences between extremities remained minimal and statistically insignificant. The observed trend of slightly lower stability scores in the contralateral extremity may indicate subtle variations in compensatory strategies or muscle recruitment. Nonetheless, these differences appear to have limited clinical relevance due to small effect sizes and the lack of statistical significance. These findings align with those presented by Alonso et al., who tested 24 male soccer players using a similar balance system and observed that the ACLR extremity performed worse than the unaffected one [25].

In the author's opinion, the most important findings concern the IST results in which significant inter-extremity differences in quadriceps and hamstring strength were found across all tests. Despite being cleared to play, the majority of the group showed moderate to large deficits in both their quadriceps and hamstring strength, as compared to the uninjured extremity. These differences were most pronounced during knee extension at both tested angular velocities. At 60 deg·s^-1^, the PT/BW ratio for the ACLR extremity was significantly lower (2.01±0.65 Nm.kg^-1^) than for the contralateral extremity (2.60±0.57 Nm.kg^-1^), with a large effect size. Similarly, at 180 deg·s^-1^, the ACLR extremity PT/BW ratio was 1.51±0.44 Nm.kg^-1^ compared to 1.88±0.35 Nm.kg^-1^ for the contralateral extremity. The LSI for the ACLR was markedly reduced, with mean values of 76.97±17.72% at 60 deg·s^-1^ and 79.89±17.11% at 180 deg·s^-1^. The study by Kublin et al. also revealed significant inter-extremity strength differences. In quadriceps, the PT/BW ratio (60 deg·s^-1^ and 180 deg·s^-1^) was 2.07±0.59 Nm.kg^-1^ and 1.56±0.44 Nm.kg^-1^ for ACLR extremity, with LSI of 75% and 79%, respectively [20]. In line with this, Herrington et al., who investigated quadriceps strength, muscle inhibition, and hop test performance in soccer players after ACLR at the time of RTS, found significant deficits in isometric, eccentric, and concentric quadriceps strength, with over 80% of players failing to meet the ≥90% LSI criterion for strength tests [26]. Normative data presented by van Melick et al. for uninjured non-elite soccer players indicate PT/BW values of 2.76±0.41 Nm.kg^-1^ for the quadriceps at 60 deg·s^-1^ and 2.05±0.20 Nm.kg^-1^ at 180 deg·s^-1^ [27]. In our study, 11 subjects (16%) achieved PT/BW values exceeding 2.76 Nm.kg^-1^ at 60 deg·s^-1^, while 15 subjects (22%) showed the LSI ≥90%. At 180 deg·s^-1^, eight subjects (12%) exceeded the PT/BW value of 2.05 Nm.kg^-1^, while 21 (30%) demonstrated an LSI ≥90%. Notably, 13 subjects (19%) achieved an LSI ≥90% at both angular velocities. While the inter-extremity differences in knee flexion were less pronounced, they still remained statistically significant with a medium effect size. At 60 deg·s^-1^, the PT/BW ratio for the ACLR extremity was 1.24±0.34 Nm.kg^-1^ vs. 1.39±0.32 Nm.kg^-1^ for the contralateral one, and at 180 deg·s^-1^, these values were 1.03±0.27 Nm.kg^-1^ and 1.16±0.25 Nm.kg^-1^, respectively. The LSI values were relatively higher, averaging 89.34±13.91% at 60 deg·s^-1^ and 88.44±14.58% at 180 deg·s^-1^. Normative data for hamstring strength indicate PT/BW values of 1.57±0.23 Nm.kg^-1^ at 60 deg·s^-1^ and 1.7±0.10 Nm.kg^-1^ at 180 deg·s^-1^ [27]. In our study, six subjects (9%) achieved PT/BW values exceeding 1.57 Nm.kg^-1^ at 60 deg·s^-1^, with 35 subjects (51%) demonstrated the LSI ≥90%. At 180 deg·s^-1^, no subjects exceeded the normative PT/BW value of 1.7 Nm.kg^-1^, while 27 subjects (39%) demonstrated an LSI ≥90%. Twenty-three subjects (33%) achieved an LSI ≥90% at both angular velocities. In summary, only three participants (4%) of the entire study group achieved an LSI ≥90% across all measured strength variables, showing the persistent asymmetry and muscle strength deficits that remain even at the point they were cleared to RTS after ACLR.

In the correlation analysis, weak but statistically significant, negative correlations were found between the FMS total score and individual stability indices for the ACLR extremity. Specifically, lower FMS scores were associated with higher (worse) OSI values on both stable and unstable platforms, as well as with the ML index on the unstable platform. This could indicate that individuals with better functional movement quality as assessed by FMS may exhibit better lower extremity control. For the contralateral extremity, correlations with stability indices were mixed and generally not significant, with only one significant result observed for the ML stability index on the unstable platform. These findings align with the broader literature, which has shown that balance ability, as quantified by tools such as the Biodex Balance System, is significantly associated with clinical knee test scores and functional performance measures following ACLR. For instance, the study by Kim et al. demonstrated that the AP index was negatively correlated with the subjective (Tegner, International Knee Documentation Committee (IKDC), and Lysholm score) and objective balance measures (single-leg hop test results and single-leg vertical jump) [28]. These results suggest that improving balance after ACLR, as reflected in both stability indices and functional test scores, may contribute to enhanced postoperative recovery in terms of functional performance. Strength assessments showed positive, significant correlations of moderate strength between the PT/BW ratios and FMS total scores, indicating that greater muscle strength was associated with a higher functional movement quality. These positive correlations ranged from 0.36 to 0.60 across various movements and velocities, reinforcing the relationship between strength and functional performance after ACLR. Additionally, the LSI for quadriceps strength demonstrated significant, moderate positive correlations with FMS scores at both 60 deg·s^-1^ and 180 deg·s^-1^ (r=0.45 and r=0.44, respectively), suggesting that better symmetry in strength between extremities may contribute to improved functional outcomes. The current findings are consistent with previous studies reporting correlations between quadriceps strength and functional performance as expressed by hop tests for distance [29] and single-leg medial rotation, single-leg lateral hop, and single-leg vertical jump tests [30].

Several limitations should be acknowledged when interpreting the findings of this study. First, the absence of preoperative data on functional outcomes, such as FMS scores, SLBT results, and muscle strength status, hindered the ability to establish baseline comparisons, which are crucial for evaluating the impact of ACLR. Second, the focus on male, non-elite soccer players restricts the generalizability of the results to other athletic populations, female athletes, or varying levels of competition. Third, while the FMS is a widely recognized tool for assessing functional movement, its predictive value for injury risk or functional capacity remains debated, with compensatory movement patterns potentially influencing scores. Fourth, the lack of normative stability index values for this specific population adds complexity to the interpretation of observed balance deficits. Lastly, although significant strength asymmetries were identified, their precise contribution to functional limitations, re-injury risk, long-term recovery outcomes, or psychological readiness for RTS were not directly assessed. The strengths of this study include the consistency of surgical procedures performed by only two surgeons with similar ACLR experiences, a standardized postoperative rehabilitation protocol, a homogeneous sport level among participants, and the use of reliable measurement tools.

## Conclusions

This study highlights the persistent neuromuscular deficits in non-elite soccer players following ACLR. While FMS scores indicated a moderate level of functional movement quality, significant inter-extremity strength asymmetries in the quadriceps and hamstring were observed. These asymmetries, particularly in quadriceps strength, emphasize the importance of incorporating targeted strength training into rehabilitation protocols. The lack of significant differences in stability indices between extremities suggests that balance alone may not be a sensitive marker of functional recovery. However, weak correlations between FMS scores and stability indices point to a relationship between functional movement quality and lower extremity balance control. Importantly, achieving only clinical clearance after ACLR does not equate to full recovery. Therefore, a comprehensive, multidimensional assessment incorporating functional performance tests, strength evaluations, balance analysis, implementation of targeted therapeutic measures, and re-evaluation should be mandatory before making an RTS decision.
